# RNA‐guided endonuclease – *in situ* labelling (RGEN‐ISL): a fast CRISPR/Cas9‐based method to label genomic sequences in various species

**DOI:** 10.1111/nph.15720

**Published:** 2019-03-08

**Authors:** Takayoshi Ishii, Veit Schubert, Solmaz Khosravi, Steven Dreissig, Janina Metje‐Sprink, Thorben Sprink, Jörg Fuchs, Armin Meister, Andreas Houben

**Affiliations:** ^1^ Leibniz Institute of Plant Genetics and Crop Plant Research (IPK) Gatersleben Seeland D‐06466 Germany; ^2^ Arid Land Research Center (ALRC) Tottori University 1390 Hamasaka Tottori 680‐0001 Japan; ^3^ Julius Kühn‐Institute Institute of Biosafety in Plant Biotechnology Quedlinburg D‐06484 Germany

**Keywords:** centromere, CRISPR/Cas9, FISH, immunostaining, *in situ* labelling, telomere

## Abstract

- Visualising the spatio‐temporal organisation of the genome will improve our understanding of how chromatin structure and function are intertwined.
- We developed a tool to visualise defined genomic sequences in fixed nuclei and chromosomes based on a two‐part guide RNA with a recombinant Cas9 endonuclease complex. This method does not require any special construct or transformation method.
- In contrast to classical fluorescence *in situ* hybridiaztion, RGEN‐ISL (RNA‐guided endonuclease – *in situ* labelling) does not require DNA denaturation, and therefore permits a better structural chromatin preservation. The application of differentially labelled *trans*‐activating crRNAs allows the multiplexing of RGEN‐ISL. Moreover, this technique is combinable with immunohistochemistry. Real‐time visualisation of the CRISPR/Cas9‐mediated DNA labelling process revealed the kinetics of the reaction.
- The broad range of adaptability of RGEN‐ISL to different temperatures and combinations of methods has the potential to advance the field of chromosome biology.

## Introduction

*In situ* hybridisation is a powerful tool to physically map DNA sequences for research and diagnostic purposes. One major disadvantage of this method is the need of any kind of DNA denaturation to achieve complementary base pairing in double‐stranded DNA (dsDNA). Such harsh treatment invariably degrades the structure of the specimen (Kozubek *et al*., 2000; Boettiger *et al*., 2016). Furthermore, classical fluorescence *in situ* hybridisation (FISH) additionally includes hybridisation and post‐hybridisation washes, which require hours to days to receive a final result.

The discovery of the type II clustered regularly interspaced short palindromic repeats (CRISPR)‐associated caspase 9 (Cas9) system derived from *Streptococcus pyogenes* as a programmable endonuclease revolutionised the field of targeted genome editing in eukaryotes (Jinek *et al*., 2012). The detection of specific sequences by CRISPR/Cas9 occurs without global DNA denaturation. First, the Cas9–RNA complex recognises a short nucleotide sequence (NGG for *S. pyogenes* Cas9) 3′ to a target sequence in the dsDNA, called a protospacer adjacent motif (PAM). Then, Cas9–RNA initiates DNA unwinding at the PAM‐proximal region, followed by the directional formation of an R‐loop, consisting of the RNA–DNA hybrid and the displaced nontarget DNA strand (Sternberg *et al*., 2014).

Cas9 nuclease‐based genome editing has become a routine technology for many species (reviewed by Ahmad *et al*., 2018). However, the full potential of this technology reaches far beyond the controlled induction of mutations (Wang *et al*., 2016). Recently, nuclease‐deficient derivatives were used to visualise genomic loci in living (Chen *et al*., 2013; Anton *et al*., 2014; Dreissig *et al*., 2017) and fixed cells (Deng *et al*., 2015). In contrast to classical FISH, CRISPR‐Cas9‐mediated *in situ* labelling of genomic loci in fixed cells does not require DNA denaturation, and therefore permits a better structural preservation of the specimen.

The use of fluorophore‐coupled single guide RNA (sgRNA) in combination with a fluorophore as well as Halo‐Tag (a bacterial hydrolase designed to covalently bind to a synthetic ligand of choice and fuse to a protein of interest) coupled nuclease‐deficient Cas9 (dCas9), termed Cas9‐mediated FISH (CAS‐FISH), allowed the labelling of repetitive DNA elements in fixed mammalian cells (Deng *et al*., 2015). Further optimisation of this technology is needed before it can be used for routine applications. In particular, the development of a technique that is simple, and does not require extensive sample preparation remains highly desirable.

Here we describe a further development of a CRISPR/Cas9‐based method for the labelling of genomic loci, which does not require the laborious *in vitro* RNA synthesis or application of a Halo‐tag approach, and therefore simplifies handling of the enzyme‐RNA complex‐based labelling of fixed plant and human nuclei and chromosomes. In addition to Cas9 from *S. pyogenes*, other RNA‐guided endonucleases also have the potential to visualise genomic loci or RNA directly (Abudayyeh *et al*., 2017). Our RGEN‐ISL method, ‘RNA‐guided endonuclease – *in situ* labelling (RGEN‐ISL)’, preserves the natural spatio‐temporal organisation of the chromatin and allows the specific and simultaneous *in situ* detection of multicoloured genomic sequences by applying a complex of a two‐part guide RNA and recombinant Cas9 endonuclease. RGEN‐ISL does not require guide RNA constructs, enzymatic *in vitro* RNA synthesis or modified Cas9 proteins. Moreover, we found that this technique is combinable with immunohistochemistry. We provide the real‐time visualisation of the CRISPR/Cas9‐mediated DNA labelling process. With its broad range of adaptability, RGEN‐ISL has the potential to advance the field of chromosome biology.

## Materials and Methods

### Material

Nuclei and chromosomes of *Nicotiana benthamiana* Domin, *Arabidopsis thaliana* (L.) Heynh, *Sorghum bicolor* (L.) Moench and *Homo sapiens* L. were used.

### Methods

#### Preparation of recombinant SpCas9

Cas9 of *S. pyogenes* encoded in vector pMJ806 (Addgene plasmid no. 39312) (Jinek *et al*., 2012) was expressed in *Escherichia coli* strain BL21 Rosetta 2 (DE3) (Novagen, Madison, WI, USA). Clarified cell lysate was bound to a 5 ml HisTrap FF (GE Healthcare, Little Chalfont, UK). After buffer exchange, the 6×His‐MBP (maltose binding protein) tag was removed by tobacco etch virus protease cleavage. Protein and cleaved fusion tag were separated on a 5 ml HiTrap SP column (GE Healthcare). After a final size exclusion chromatography step on a HiLoad 16/600 Superdex 200 column (GE Healthcare) in 30 mM Hepes, pH 7.5, 200 mM NaCl, 2 mM MgCl_2_ and 1 mM TCEP, the protein was concentrated to *c. *6 mg ml^−1^ using the Pierce Protein Concentrator PES, 30k molecular weight cut‐off according to the instructions of the manufacturer, flash frozen in liquid nitrogen and stored at −80°C.

#### Formation of the ribonucleoprotein

We selected the two‐part guide RNA (crRNA and *trans*‐activating crRNA (tracrRNA)) system (Alt‐R CRISPR‐Cas9) for the production of functional guide RNA commercially prepared by the company IDT (Integrated DNA Technologies, https://eu.idtdna.com) (Jacobi *et al*., 2017). For guide RNA formation, lyophilised crRNA and tracrRNA‐ATTO 550/Alexa Fluor 488 was prepared in nuclease‐free duplex buffer (30 mM Hepes, pH 7.5; 100 mM CH_3_COOK) to 100 μM. Dissolved crRNA and tracrRNA can be stored separately at −20°C. crRNA, ATTO 550‐labelled tracrRNA (Alt‐R CRISPR‐Cas9 system) and nuclease‐free duplex buffer are products of IDT. Then, 20 mer of target specific sequences were selected based on the respective PAM sequence of *S. pyogenes* Cas9 with the help of the web base tool crisprdirect (https://crispr.dbcls.jp/) (Naito *et al*., 2015) (Supporting Information Table S1). Target‐specific crRNAs were purchased through the IDT website according to IDT instructions. To assemble 10 μM guide RNA : 1 μl 100 μM crRNA, 1 μl 100 μM tracrRNA‐ATTO 550/Alexa Fluor 488 was mixed with 8 μl duplex buffer. Then, the guide RNA (gRNA) was denatured for 5 min at 95°C and allowed to hybridise. gRNA is stable at −20°C until use. For ribonucleoprotein complex (RNP) complex assembly, 1 μl 10 μM guide RNA, 1 μl 6.25 μM dCas9 proteins (D10A and H840A; cat. no.: PR‐137213, Novateinbio, www.novateinbio.com), 10 μl 10×Cas9 buffer (200 mM Hepes (pH 7.5), 1 M KCl, 50 mM MgCl_2_, 50% (v/v) glycerol, 10% bovine serum albumin (BSA) and 1% Tween 20), 10 μl 10 mM dithiothreitol (DTT) and 80 μl double distilled water were mixed and incubated at 26°C for 10 min, and stored at 4°C. A volume of 100 μl of the RNP complex was sufficient for 3–4 slides. To increase the range of future RGEN‐ISL applications we tested different types of Cas9 proteins. Instead of dCas9, the following Cas9 variants can be used: recombinant SpCas9 with MBP‐tag (maltose‐binding protein) or without MBP‐tag described above, active Cas9 (Alt‐R S.p. Cas9 Nuclease, 3NLS) and Nickase Cas9 (Alt‐R S.p. Cas9 D10A Nickase, IDT).

#### Preparation of nuclei and chromosomes for RGEN‐ISL

Leaf and root tissues were fixed in ice cold 4% formaldehyde in Tris buffer (10 mM Tris‐HCl (pH 7.5), 10 mM Na_2_‐EDTA, 100 mM NaCl, 0.1% Triton X‐100 (adjusted to pH 7.5 with NaOH) for 5 min under vacuum. The fixation was continued for 25 min in ice cold fixative without vacuum. Tissues were rinsed twice for 5 min in ice cold Tris buffer. Then, the tissue was chopped in 400–500 μl ice cold LB01 buffer as described (Doležel *et al*., 1989) (LB01‐buffer: 15 mM Tris‐HCl (pH 7.5), 2 mM Na_2_‐EDTA, 0.5 mM Spermin, 80 mM KCl, 20 mM NaCl, 15 mM β‐mercaptoethanol, 0.1% Triton X‐100). The suspension was filtered through a mesh of 35 μm pore size and spun onto glass slides using a cytospin (700 rpm for 5 min). Subsequently, the slides were kept in ice cold 1×PBS until use.

#### RGEN‐ISL procedure

For each slide, 100 μl of 1×Cas9 buffer/1 mM DTT (10×Cas9 buffer: 200 mM Hepes (pH 7.5), 1 M KCl, 50 mM MgCl_2_, 50% (v/v) glycerol, 10% BSA and 1% Tween 20) was applied for 2 min at room temperature. The slides were then tilted to remove the buffer and 20–30 μl RNP complex per slide was added. Slides were covered with parafilm and kept in a moisture chamber at 26°C for 1–4 h. Note that the incubation time was species‐ and sequence‐dependent. In addition, incubation at 4°C overnight was possible. After incubation slides were washed in ice cold 1×PBS for 5 min. In case there were strong background signals an extended washing time was needed. To prevent dissociation of the RNP complex, post‐fixation was performed with 4% formaldehyde in 1×PBS for 5 min on ice. Then, slides were washed with 1×PBS for 5 min on ice and dehydrated with ethanol (70%, 90%, 96%; 2 min each) at room temperature. Slides were counterstained with 4′,6‐diamino‐2‐phenylindole (DAPI) and mounted in Vectashield mounting medium (Vector Laboratories, Burlingame, CA, USA).

#### Combination of immunostaining and RGEN‐ISL

The slides were prepared as described for RGEN‐ISL. A volume of 60–70 μl of primary antibodies (e.g. anti‐AtCENH3 rabbit polyclonal antibody) diluted 1 : 1000 in 1×PBS (Talbert *et al*., 2002) was applied per slide, and incubated at 4°C overnight in a moist chamber. Slides were washed in 1×PBS twice for 5 min on ice. Then, 100 μl of 1×Cas9 buffer/1 mM DTT was applied per slide for 2 min at room temperature. Afterwards, the buffer was replaced by a buffer (1×Cas9 buffer/1 mM DTT) containing the secondary antibodies (e.g. goat anti‐rabbit IgG Alexa488, Thermo Fisher, diluted 1 : 500) containing the prepared RNP complex. Then, 20–30 μl of the RNP complex including the diluted secondary antibody was applied per slide. Slides were covered with parafilm and kept in a moist chamber at 37°C for 1 h. Subsequently, the slides were washed in ice cold 1×PBS for 5 min. Post‐fixation and subsequent steps were performed as described for RGEN‐ISL.

#### Transient transformation of *N. benthamiana*

The Sp‐dCas9‐eGFP construct to label *Arabidopsis*‐type telomeres (Dreissig *et al*., 2017) and TRB1‐GFP (Schrumpfová *et al*., 2014) were transformed separately into *Agrobacterium tumefaciens* strain GV3101 by electroporation. The transient transformation of *N. benthamiana* leaf cells was performed as described by Phan & Conrad (2016). Nuclei for subsequent RGEN‐ISL were isolated 2–4 days after infiltration and used for RGEN‐ISL.

#### Microscopy

Classical fluorescence imaging was performed using an Olympus BX61 microscope equipped with an ORCA‐ER CCD camera (Hamamatsu). All images were acquired in grey scale and pseudocoloured with Adobe Photoshop 6 (Adobe Systems). To analyse the ultrastructure and spatial arrangement of signals and chromatin at a lateral resolution of *c*. 120 nm (super‐resolution, achieved with a 488 nm laser), three‐dimensional structured illumination microscopy (3D‐SIM) was applied using a Plan‐Apochromat 63×/1.4 oil objective of an Elyra PS.1 microscope system and the software zenblack (Carl Zeiss GmbH). Image stacks were captured separately for each fluorochrome using 561, 488 and 405 nm laser lines for excitation and appropriate emission filters (Weisshart *et al*., 2016). Maximum intensity projections of whole nuclei were calculated via the zen software. Magnified sections were presented as single slices to indicate the subnuclear chromatin and protein structures at the super‐resolution level.

Wide‐field time‐lapse videos were acquired with the same Elyra PS.1 system and processed by the imaris 8.0 (bitplane) software. Telomere signal intensity measurements were performed using the zen software tool profile.

For time‐lapse microscopy, 15 μI of isolated *N. benthamiana* nuclei were mixed with 15 μl of sucrose buffer (50 mM KCl, 2 mM MgCl_2_, 10 mM Tris‐HCl, 0.05% Tween 20, 5% sucrose, adjusted to pH 7.5 with KOH) on a coverslip (22 × 22 mm) and kept at room temperature until dry. The coverslips were placed into a Chamlide coverslip chamber (Live Cell Instrument, Seoul, Korea, cat. no. CM‐S22‐1), and washed in 1×PBS for 5 min at room temperature. After removing 1×PBS from the coverslip, 200 μl of the RNP complex was applied to the coverslip and the time‐lapse microscopy was immediately started afterwards.

### Statistics

For statistical calculation the program package sigmastat 12.0 was used (Systat Software Inc., Chicago, IL, USA). We used the two‐tailed Student's *t*‐test for comparison of two samples and one‐way ANOVA followed by pairwise comparison for more than two samples. In each case we checked the data for normality and equal variance. Error bars represent the 95% confidence interval (CI) or standard errors of the mean (SE) as indicated.

## Results and Discussion

### RGEN‐ISL *–* an *in situ* method to label specific genomic sequences specifically

**Figure 1:**
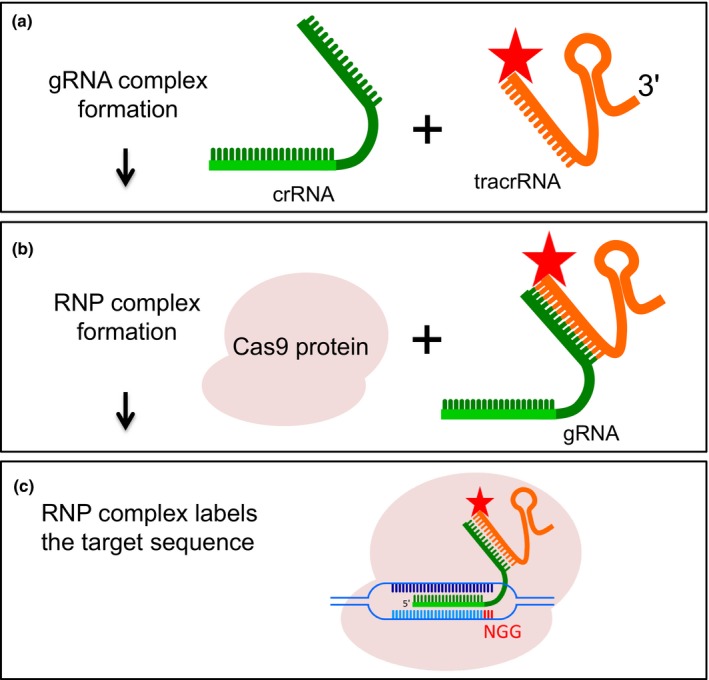
Schemata of the RNA‐guided endonuclease – *in situ* labelling (RGEN‐ISL) method. (a) Guide RNA (gRNA) complex formation after hybridisation of CRISPR RNA (crRNA) and 5′ ATTO 550 (star) labelled *trans*‐activating crRNA (tracrRNA). (b) Ribonucleoprotein (RNP) complex formation after combination of the recombinant Cas9 protein with gRNA. (c) Components of the RGEN‐ISL system to label genomic targets. The crRNA : tracrRNA complex uses optimised Alt‐R crRNA and ATTO 550‐labelled tracrRNA sequences that hybridise, and then form a complex with Cas9 endonuclease to guide targeted binding to genomic DNA. The binding site is specified by the protospacer element of the crRNA (light green bar). The crRNA protospacer element recognises 19 or 20 nt on the opposite strand of the NGG protospacer adjacent motif (PAM) site. The PAM site (red) must be present immediately downstream of the protospacer element for binding to occur.

To produce a fluorescently labelled RNP, we employed recombinant dCas9 protein of *S. pyogenes*. The mature guide RNA is naturally composed of two types of RNA molecules, a target‐specific crisprRNA (crRNA, 42 nt) and a *trans*‐activating crRNA (tracrRNA, 89 nt) (Jinek *et al*., 2012). Alternatively, the mature guide RNA can be composed of a single molecule, fused crRNA and tracrRNA combined by a 3 bp (GAA) linker, called single guide RNA (sgRNA) (Jinek *et al*., 2012). We selected the two‐molecule guide RNA system for the production of mature guide RNAs (Alt‐R system, an IDT product) (Jacobi *et al*., 2017). Because the synthesis of fluorochrome‐coupled sgRNA probes (*c*. 100 nt) is costly, we decided to test a universally 5′ ATT 550‐labelled 67 nt Alt‐R tracrRNA (Schubert *et al*., 2017). The synthetic 67 nt Alt‐R tracrRNA and the 36 nt Alt‐R‐crRNA are much shorter than the native guide RNA of *S. pyogenes* (89/42 nt) and contain proprietary chemical modifications conferring increased nuclease resistance leading to an increased on‐target performance and easy handling (Jacobi *et al*., 2017). Alt‐R tracrRNA hybridises to the 16 nt complementary region of the crRNA to activate the Cas9 enzyme. The 35–36 nt crRNA, containing the 19 or 20 nt target‐specific protospacer element, recognises the complementary strand of the NGG PAM site. The Alt‐R tracrRNA pairs with the target‐specific crRNAs, thus allowing the easy and cost‐effective study of many gRNA‐specific sites (Fig. 1).

**Figure 2:**
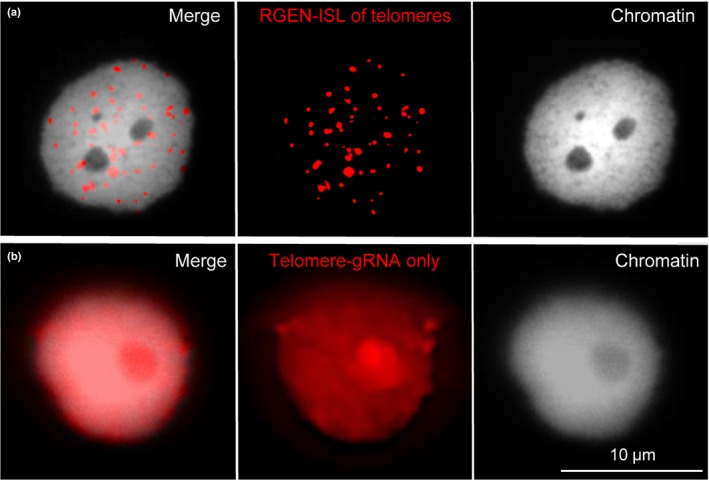
RNA‐guided endonuclease – *in situ* labelling (RGEN‐ISL) with an *Arabidopsis*‐type telomere‐specific guide RNA. (a) Nucleus of *Nicotiana benthamiana* showing telomere‐typical dot‐like signals distributed within the entire nucleus. (b) Application of ATTO 550‐labelled guide RNA without the Cas9 protein as a negative control.

To assay the functionality of RGEN‐ISL, we imaged the telomeres in formaldehyde‐fixed nuclei of *N. benthamiana*. These telomeres are composed of 60–160 kb‐long arrays of TTTAGGG repeats (Fajkus *et al*., 1995). These tandemly repeated DNA sequences allow the binding of many Cas9 proteins at the same locus by a single crRNA sequence. To target telomeric repeats, we selected a 20 nt‐long crRNA complementary to the TTTAGGG telomere sequence starting with a ‘G’ at the 5′‐end, as we had previously used for live cell CRISPR imaging (Dreissig *et al*., 2017). Bright fluorescence signals were observed within the nuclei (Fig. 2a). Performing the same experiment without the Cas9 protein as a negative control resulted in only a diffuse unspecific labelling of the entire nucleus (Fig. 2b).

**Figure 3:**
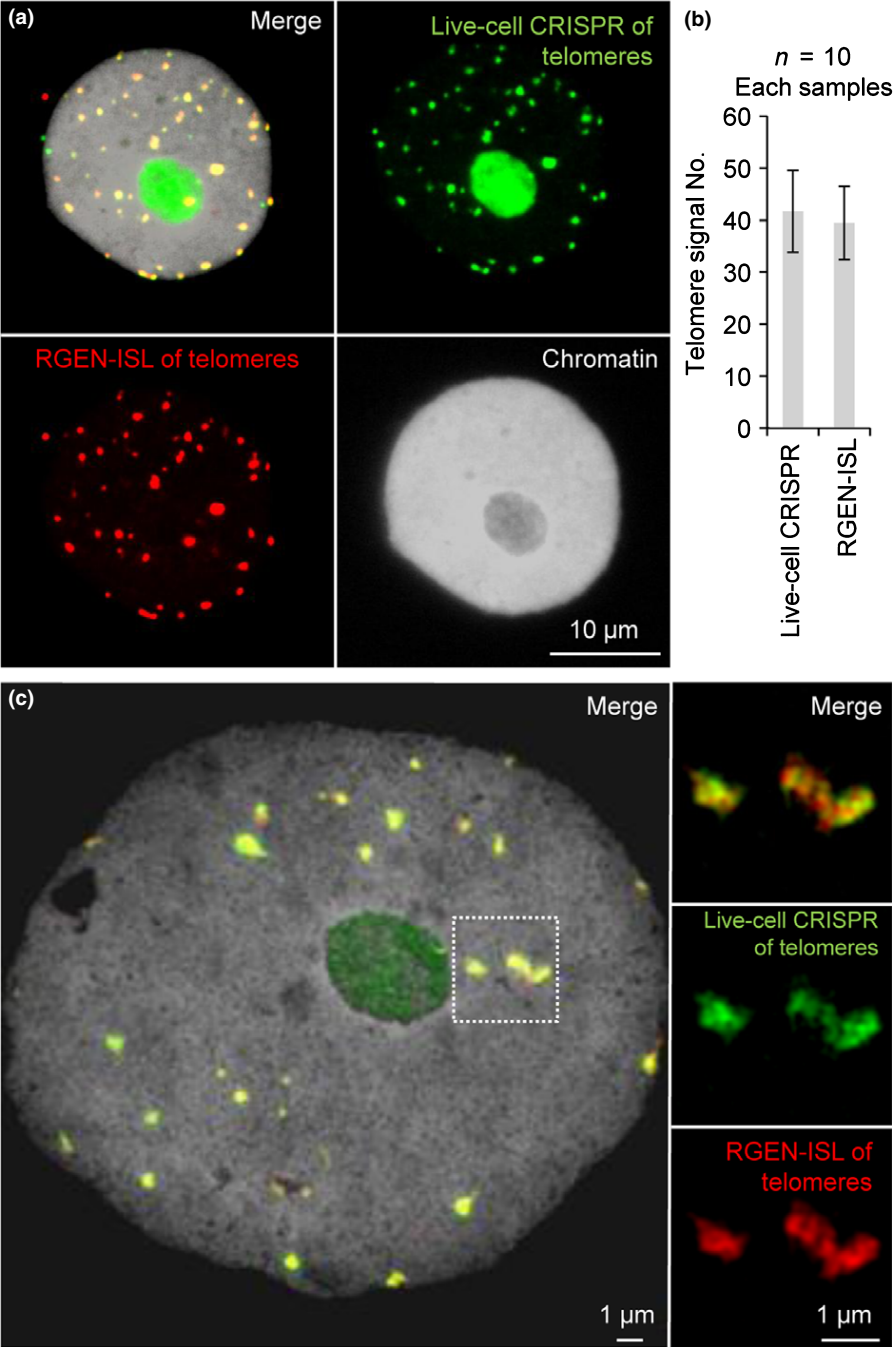
RNA‐guided endonuclease – *in situ* labelling (RGEN‐ISL) specifically detects the sequence of interest. (a) Nucleus of *Nicotiana benthamiana* exhibiting telomere‐specific live‐cell CRISPR imaging signals after subsequent telomere‐specific RGEN‐ISL. (b) Numbers of telomere live‐cell CRISPR and REGEN‐ISL signals per nucleus (*N *=* *10 nuclei) were not significantly different (*t *=* *0.409, df = 18, *P *=* *0.69, error bars represent confidence intervals). (c) Structured illumination microscopy (SIM) demonstrates a similar arrangement of live‐cell CRISPR imaging and RGEN‐ISL signals. Insets show a further enlarged subnuclear region exhibiting the telomere signal shapes.

To confirm the specificity and efficiency of the RGEN‐ISL method we repeated our experiments on nuclei of *N. benthamiana* exhibiting telomere‐specific dCas9::GFP signals (Dreissig *et al*., 2017). A clear overlap of live‐cell CRISPR imaging (telomere‐specific dCas9::GFP transient signals) and RGEN‐ISL telomere signals was found (Fig. 3a,b). However, instead of the potentially up to 76 expected telomere signals (2*n* = 38 chromosomes), on average only 39.4 telomere RGEN‐ISL signals (*N* = 10 in each sample) were found after combining live‐cell CRISPR imaging and RGEN‐ISL. Using conventional FISH on average 35 telomere signals were counted in *N. benthamiana* leaf nuclei (Dreissig *et al*., 2017). The discrepancy between expected and observed signal number suggests that some telomeres are below the threshold of detectability using these approaches. In addition, a clustering of individual telomeres cannot be excluded completely, as found in *A. thaliana* (Schubert *et al*., 2012; Fujimoto *et al*., 2016).

To determine the subnuclear localisation of both types of RNA‐guided endonuclease‐based DNA imaging probes, we used SIM to achieve a resolution of up to 120 nm. We found a similar arrangement and intensity of RGEN‐ISL and live‐cell CRISPR‐imaging signals (Fig. 3c). Hence, both methods have a similar labelling efficiency. The ability of RGEN‐ISL to label the same genomic loci, which were already detected by live‐cell CRISPR‐imaging, suggests that only a subset of telomere repeats per chromosome end was detected by the live‐cell CRISPR imaging reporter.

**Figure 4:**
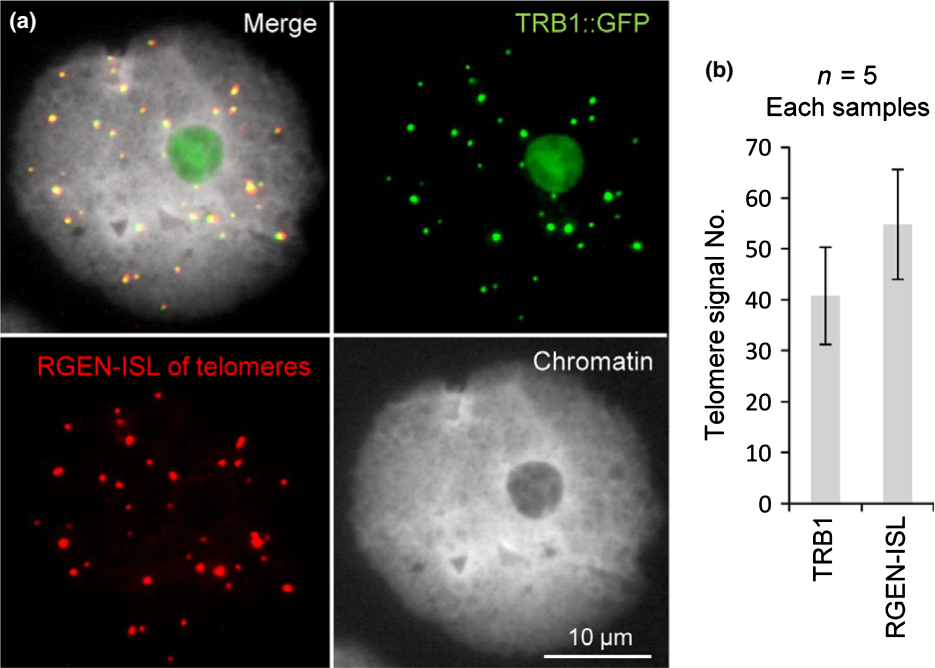
RNA‐guided endonuclease – *in situ* labelling (RGEN‐ISL) in combination with fluorescently tagged proteins allows the double labelling of DNA and endogenous proteins. (a) Nucleus of *Nicotiana benthamiana* exhibiting TRB1::GFP signals after subsequent telomere‐specific RGEN‐ISL. (b) Numbers of TRB1::GPF and telomere‐specific RGEN‐ISL signals per nucleus (*N *=* *5 nuclei) were not significantly different (*t *=* *1.904, df = 8, *P *=* *0.093, error bars represent confidence intervals).

In addition, we labelled fixed nuclei expressing the GFP‐fused telomere repeat binding protein 1 (TRB1) (Fig. 4). TRB1 locates at telomeres and interacts with the telomere reverse transcriptase, although not all telomeres are bound by TRB1 (Dvorácková *et al*., 2010; Schrumpfová *et al*., 2014). On average, we detected 50 RGEN‐ISL signals resembling telomeres, of which 40 (87.6%) were simultaneously bound by TRB1 (Fig. 4). Our results demonstrate that RGEN‐ISL can also be used to visualise specific DNA sequences in combination with fluorescently tagged endogenous proteins interacting with those DNA sequences.

**Figure 5:**
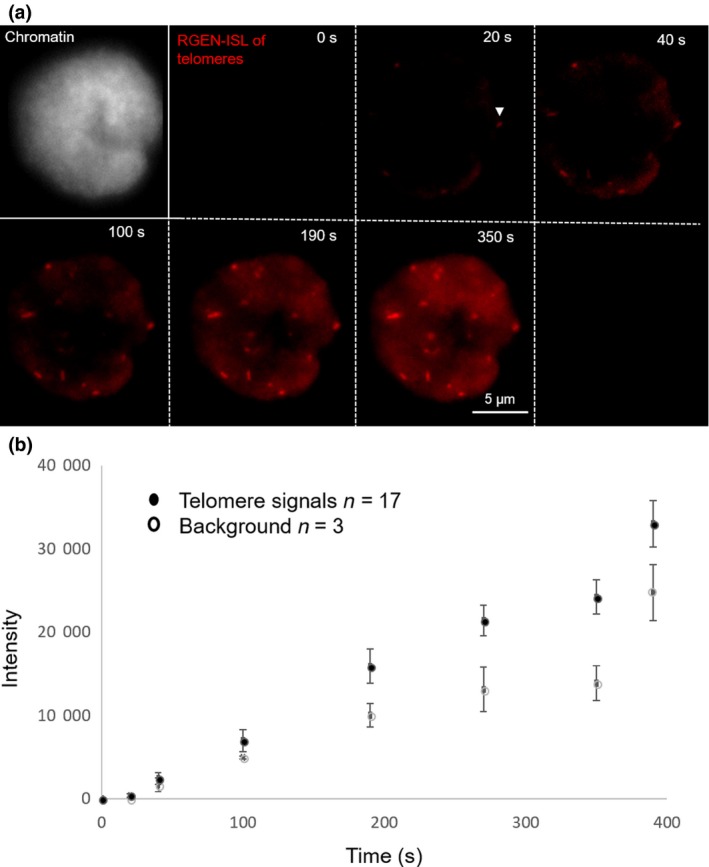
Time‐lapse microscopy demonstrates the dynamics of the dCas9–RNA complex to label the telomeres in fixed *Nicotiana benthamiana* nuclei. (a) First telomere signals (white arrowhead) are visible 20 s after applying the ribonucleoprotein (RNP) complex. Nucleus is counterstained with 4′,6‐diamino‐2‐phenylindole (DAPI). (b) The telomere as well as the background increase in the course of time. The intensities of 17 telomere signals at eight different time points were measured from 0 to 390 s. In parallel, we determined the background intensity at three different sites outside the telomeres. For each time point we calculated the mean ± SE.

The Cas9–RNA complex interrogates the target sites on the DNA via 3D diffusion, and recognises the complementary target site with the NGG PAM diffusion (Shibata *et al*., 2017). We used time‐lapse microscopy to visualise the real‐time dynamics of the dCas9–RNA complex for the labelling of telomeres of fixed nuclei in action (Fig. 5, Video S1, Fig. S1). Notably, just 20 s after applying the pre‐assembled dCas9–RNA complex, the first telomere signals became detectable. The intensity of fluorescence signals increased steadily over the next 390 s. However, in parallel background signals also increased, as no washing step was included. The increase of the telomere signal intensity suggests, in line with previous biochemical experiments (Sternberg *et al*., 2014), that the dCas9–RNA complex remains tightly bound to the DNA after target identification.

We noted that incubation temperatures between 4 and 37°C are suitable for this method and we chose to use 26°C for most experiments (Fig. S2). To maintain the fluorescence signals after the RGEN‐ISL reaction, a short post‐fixation step in 4% formaldehyde on ice is required to prevent dissociation of the dCas9–RNA complex from the DNA. Besides the application of the nuclease‐deficient dCas9 protein, recombinant active Cas9, Cas9 with an MBP‐tag or Cas9 nickase were also successfully used for RGEN‐ISL (Fig. S3a,b). Irrespective of the cutting ability, all Cas9 variants resulted in a similar number (no significant difference, *F*_3,24_ = 0.35, *P* = 0.79) and intensity of telomere signals after a fixation of 5 min. For most of the RGEN‐ISL experiments, we used a 5 min 4% formaldehyde fixation under vacuum. On average 60 telomere signals were found per nucleus (*N* = 7). As the combination of live‐cell CRISPR imaging and RGEN‐ISL resulted in the detection of 39.4 telomere signals only (Fig. 3), it is likely that pre‐occupation of telomere sites with the ‘live‐cell CRISPR imaging RNA/protein complex’ is reducing the labelling efficiency of the RGEN‐ISL. An extended formaldehyde‐based fixation (10 min) of tissues reduced the efficiency of the RGEN‐ISL method, probably by blocking the ribonucleoprotein complex access to the target sequence (Fig. S3a,b). The same observation was made for ethanol/acetic acid‐fixed tissue, a method that is routinely used for the preparation of chromosomes and nuclei.

By contrast, methanol/acetic acid‐based fixation of tissue as used by Deng *et al*. (2015) leads to poor morphological preservation of nuclei because the fixation time (−20°C for 20 min) was not sufficient to fix the plant tissue properly (Fig. S4). The application of unfixed nuclei resulted in the strongest RGEN‐ISL signals, but was accompanied by destructed nuclei. Furthermore, the exclusive fixation of tissues in glyoxal only (Richter *et al*., 2018) did not perform better than formaldehyde (Fig. S4). Each tissue type and species probably requires an optimisation of the fixation method suitable for RGEN‐ISL.

**Figure 6:**
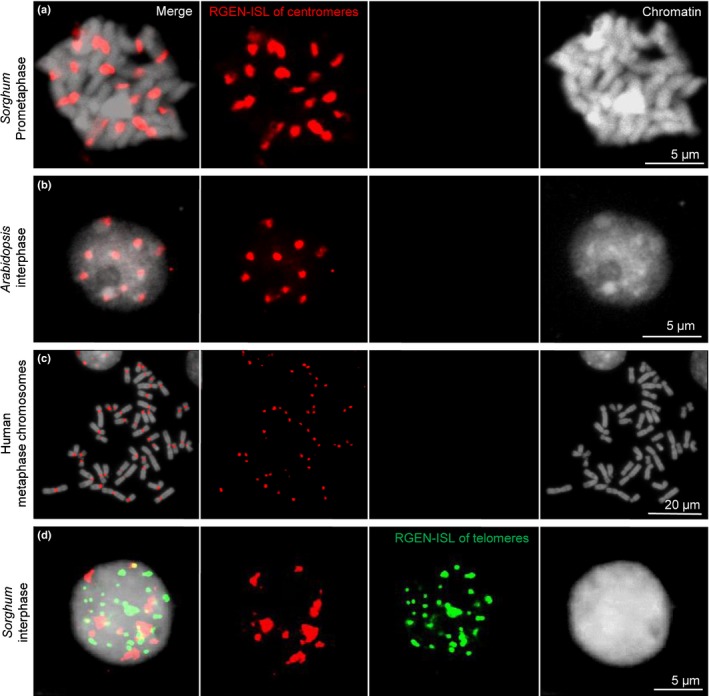
RNA‐guided endonuclease – *in situ* labelling (RGEN‐ISL) for centromeres in different species and multicolour RGEN‐ISL. (a–c) RGEN‐ISL‐based detection of centromere repeats in *Sorghum bicolor*,* Arabidopsis thaliana* and *Homo sapiens*. (d) Multicolour RGEN‐ISL allows the simultaneous detection of telomere and centromere repeats in *S. bicolor*.

Beside the detection of telomeres, we demonstrated that RGEN‐ISL allows the detection of centromeric tandem repeats in *S. bicolor* (Miller *et al*., 1998), *A. thaliana* (Murata *et al*., 1994) and *H. sapiens* (Vissel & Choo, 1987) (Fig. 6a–c, respectively). Furthermore, the simultaneous application of 5′ Alexa fluor 488 and 5′ ATTO 550 labelled tracRNAs paved the way to differentially label multiple genomic loci including the centromeres and telomeres of *S. bicolor* (Fig. 6d).

**Figure 7:**
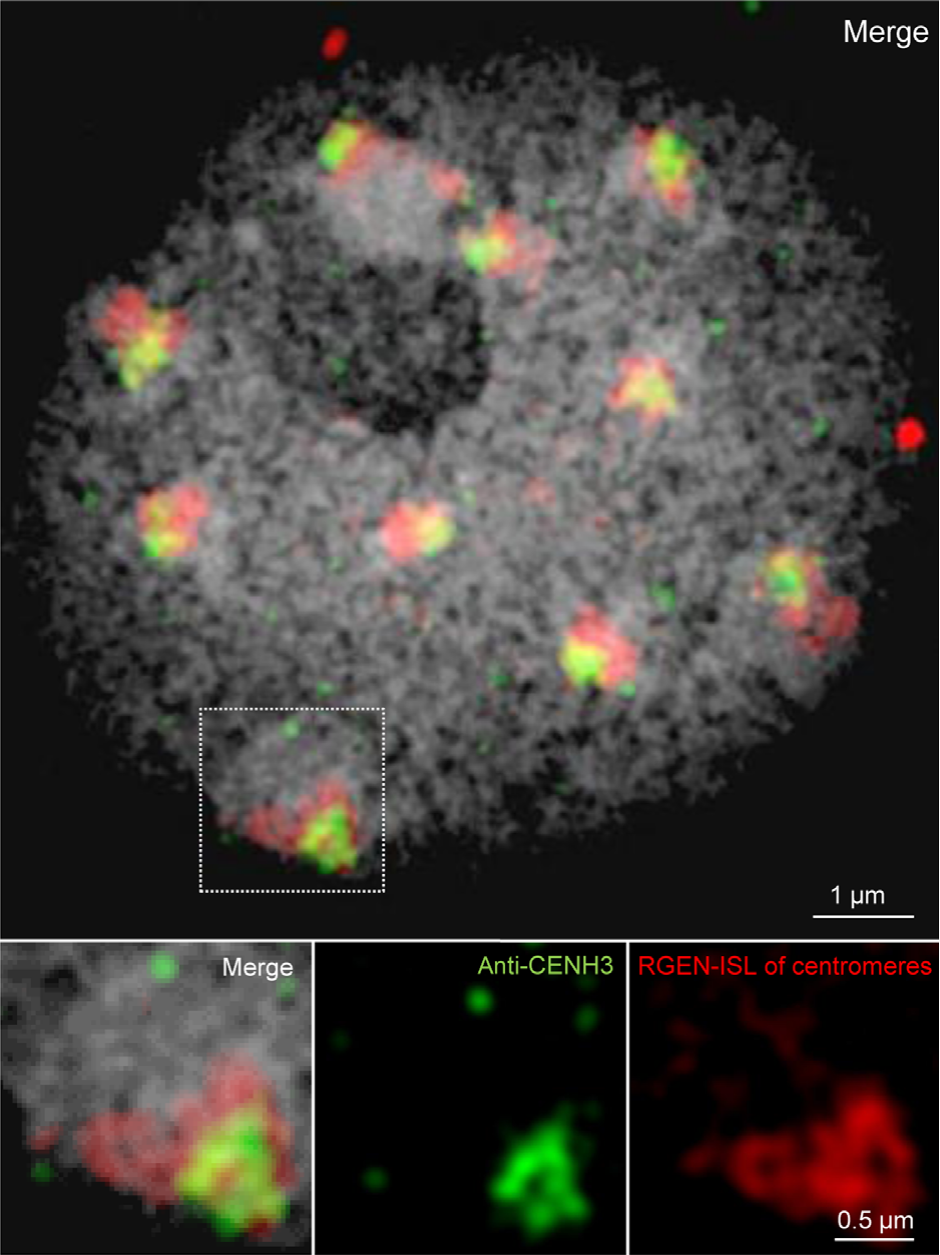
RNA‐guided endonuclease – *in situ* labelling (RGEN‐ISL) in combination with immunostaining allows the simultaneous labelling of DNA and endogenous proteins. Arabidopsis interphase nucleus showing anti‐CENH3 and centromere‐specific RGEN‐ISL signals. The insets show a further enlarged subnuclear region exhibiting centromeric signals.

Because RGEN‐ISL allows us to visualise specific DNA sequences in nondenatured chromatin, it can be combined with protein‐based detection methods, such as immunofluorescence, to study the colocalisation of DNA–protein complexes. As an example, we visualised simultaneously the centromeric DNA by RGEN‐ISL and the centromere‐specific histone H3 variant CENH3 by immunostaining in *A. thaliana* nuclei (Fig. 7). Fast detection of specific DNA and proteins is possible, as RGEN‐ISL and immunostaining work in the same buffer. We hypothesise that this principle can be expanded to investigate spatio‐temporal gene expression patterns, for example by visualising DNA sequences and histone variants.

One limitation of RGEN‐ISL is the requirement of the presence of a PAM at the target site. For the most commonly used Cas9 from *S. pyogenes*, the required PAM sequence is NGG. To overcome this limitation, natural or engineered Cas9 variants with different PAM sequence requirements could be used (Ma *et al*., 2015; Hu *et al*., 2018). For the detection of tandem repetitive sequences, a single guide RNA is sufficient for RGEN‐ISL imaging. However, the detection of nonrepetitive loci may require simultaneous application of multiple guide RNAs, as demonstrated for the CRISPR‐based detection of a 5 kb region in living mammalian cells (Chen *et al*., 2013). Further developments may improve the CRISPR‐based DNA detection and potentially enable us to visualise even single genomic loci.

## Author contributions

TI conceived and designed the study, performed experiments, analysed data and wrote the manuscript. VS, SK, SD, JM‐S, TS, JF and AM performed experiments, analysed data and assisted with preparing the manuscript. AH conceived the study, performed experiments and wrote the manuscript.

## Supporting information

Please note: Wiley Blackwell are not responsible for the content or functionality of any Supporting Information supplied by the authors. Any queries (other than missing material) should be directed to the *New Phytologist* Central Office.

**Fig. S1** Time‐lapse microscopy demonstrates a similar tendency of telomere and background signals increasing pattern in fixed *N. benthamiana* nuclei.
**Fig. S2** An incubation temperature of 4–37°C is suitable for RGEN‐ISL.
**Fig. S3** Comparison of different types of Cas9 and fixation conditions for RGEN‐ISL.
**Fig. S4** Influence of the fixation method on RGEN‐ISL‐based telomere signals.
**Table S1** crRNA sequences used for RGEN‐ISL.

**Video S1** Time‐lapse microscopy showing the dynamics of the dCas9–RNA complex to label the telomeres of fixed *N. benthamiana* nuclei.
