# Supplementary material for: RNA‐guided endonuclease – in situ labelling (RGEN‐ISL): a fast CRISPR/Cas9‐based method to label genomic sequences in various species
Source: New Phytol. 2019 Mar 8;222(3):1652–61. doi: 10.1111/nph.15720 (PMC6593734; doi:10.1111/nph.15720)
Supplement: Supplementary file 1 — Fig. S1 Time‐lapse microscopy demonstrates a similar tendency of telomere and background signals increasing pattern in fixed N. benthamiana nuclei. Fig. S2 An incubation temperature of 4–37°C is suitable for RGEN‐ISL. Fig. S3 Comparison of different types of Cas9 and fixation conditions for RGEN‐ISL. Fig. S4 Influence of the fixation method on RGEN‐ISL‐based telomere signals. Table S1 crRNA sequences used for RGEN‐ISL. [file NPH-222-1652-s001.pdf]

New Phytologist

***New Phytologist* Supporting Information**

Article title: RNA-guided endonuclease - in situ *labelling (*RGEN-ISL) - a fast CRISPR/Cas9 based method to label genomic sequences in various species

Authors: Takayoshi Ishii, Veit Schubert, Solmaz Khosravi, Steven Dreissig, Janina Metje-Sprink, Thorben Sprink, Jörg Fuchs, Armin Meister and Andreas Houben

Article acceptance date: 24 January 2019

The following Supporting Information is available for this article:

New Phytologist

Fig. S1 Time-lapse microscopy demonstrates similar tendency of the telomere and background signal increasing pattern in fixed *N. benthamiana* nuclei**.** The telomere as well as the background signals similarly increase over time in different nuclei from Fig. 5. The intensities of 12 telomere signals at 8 different time points from 0 to 390 seconds were measured. In parallel the background intensity was determined at 3 different sites outside the telomeres. For each time point we calculated the mean and standard error of mean. Mean telomere signal (●) and background (○) intensities ± standard errors of mean. The data originate from another nucleus than that shown in fig. 5, but confirm the same tendency.

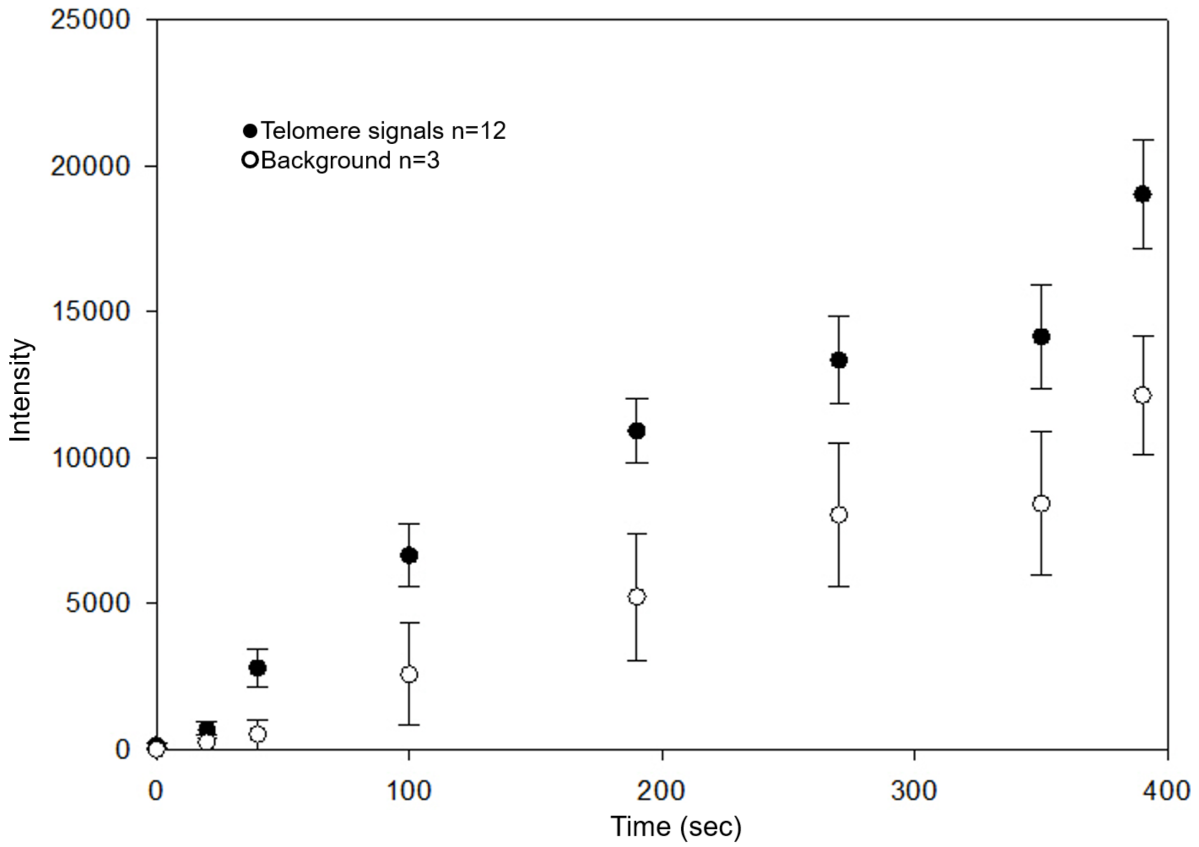

New Phytologist

Fig. S2 An incubation temperature between 4°C - 37°C is suitable for RGEN-ISL. Telomere-specific RGEN-ISL was performed under identical conditions (1 hour incubation), except different temperatures (4°C, 26°C and 37°C). The telomere signals of *N. benthamiana* nuclei were recorded with the same CCD camera setting (200 msec exposure time in all cases).

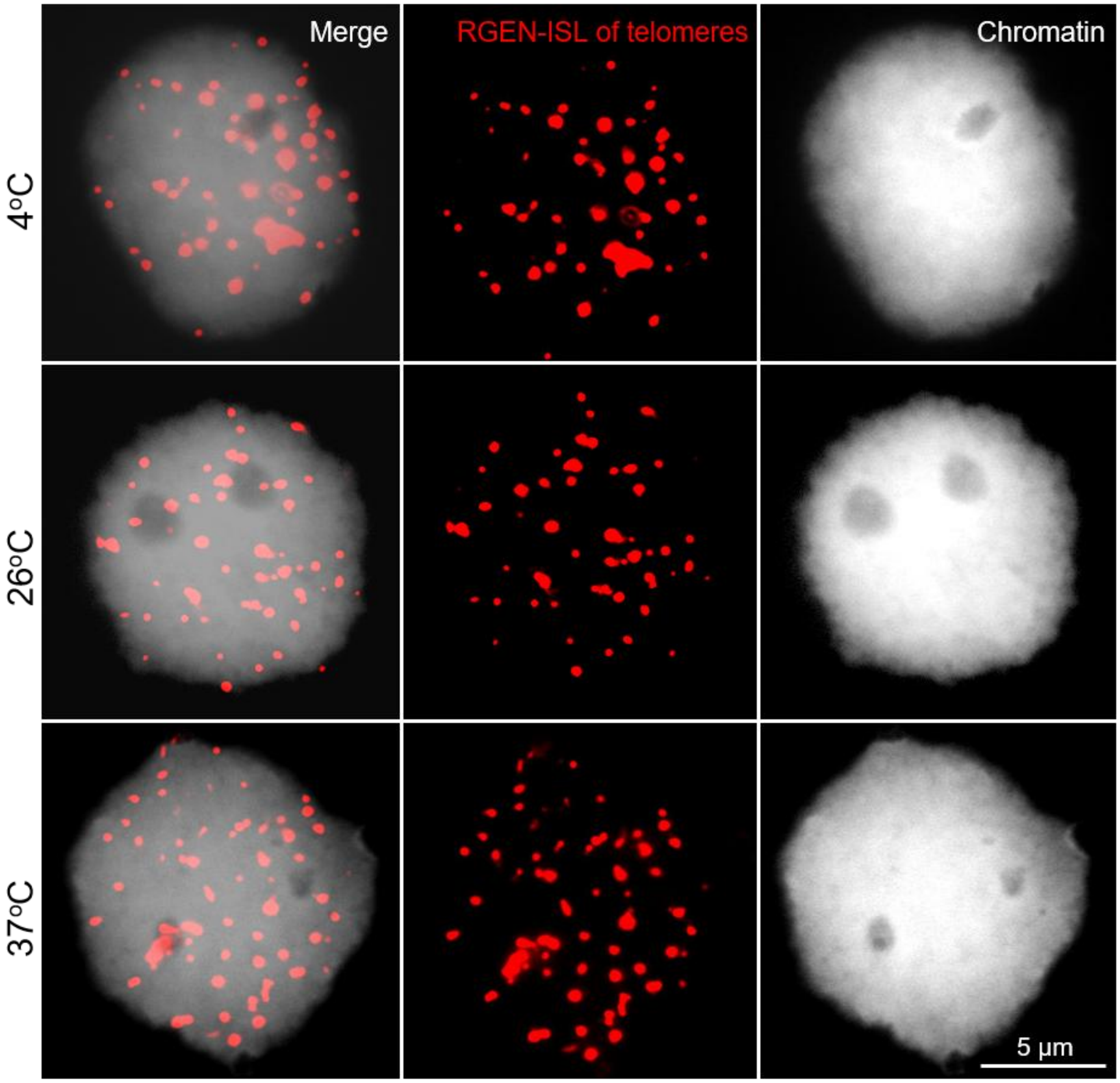

Fig. S3 Comparison of different types of Cas9 and fixation condition for RGEN-ISL. (a) Nuclei of *N. benthamiana* showing telomere signals irrespective of the cutting activity of the Cas9 (nuclease-deficient Cas9 (dCas9), active Cas9, Cas9 with a MBP-tag or Cas9 nickase) by 5 minutes 4% formaldehyde fixation under vacuum. (b) Telomere signals are significantly reduced when using a 10 minute vacuum with 4% formaldehyde-fixation (F(4;30)=41.3,; P<0.001/***, Error bars represent C. I.).

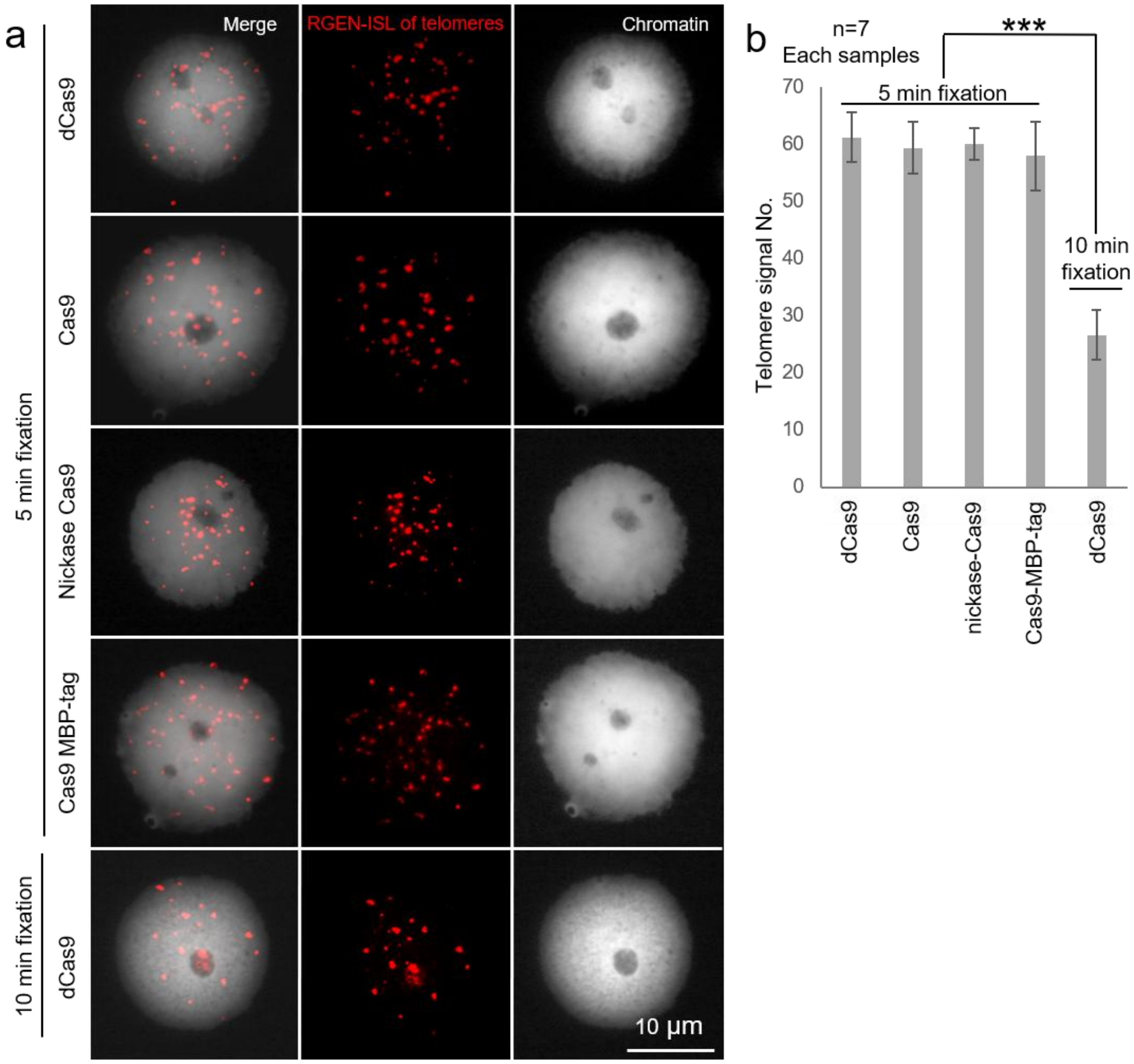

Fig. S4 Influence of fixation method on RGEN-ISL based telomere signals. Before nuclei isolation and RGEN-ISL, leaf tissues of *N. benthamiana* were fixed with either -20°C prechilled solution of methanol/acetic acid (1:1), 2% solved paraformaldehyde (PFA), 4% solved paraformaldehyde (PFA) or glyoxal (Richter *et al.*, 2018). Note, the unfixed tissue resulted in the strongest RGEN-ISL signals.

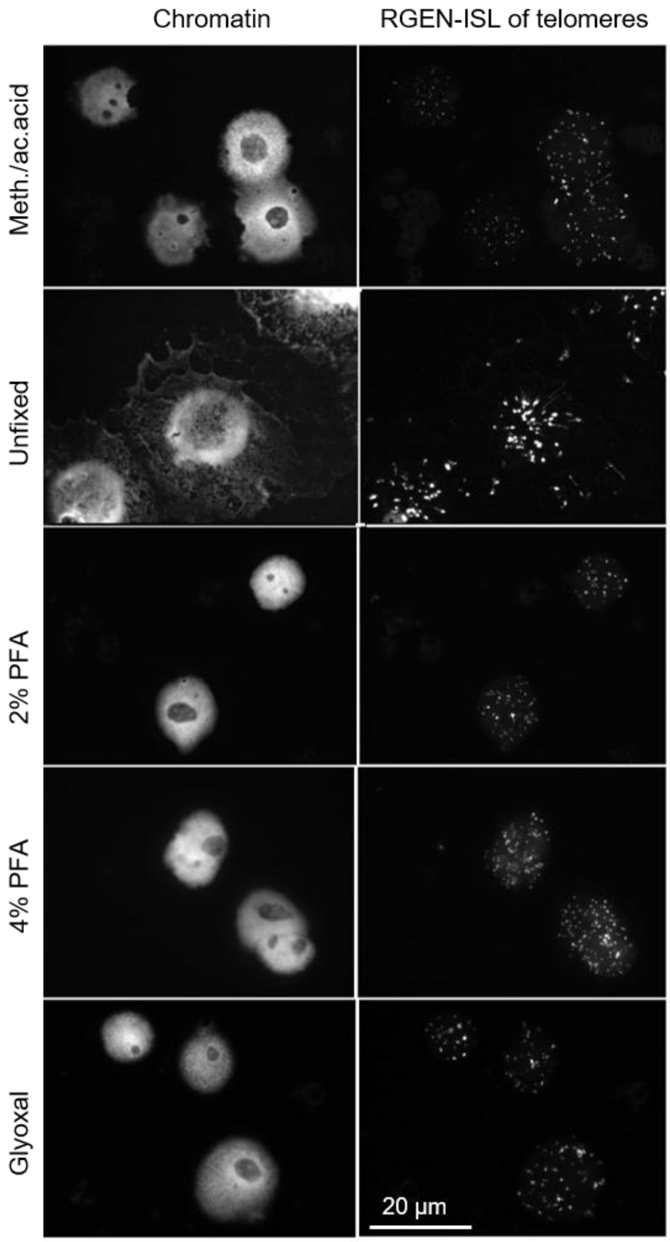

New Phytologist

Table S1 crRNA sequences used for RGEN-ISL.

Name	Sequence
Sorghum centromere	5'-ATTAGGATAAGAAACCATTT-3'
Arabidopsis-type telomere	5'-GGGTTTAGGGTTTAGGGTTT-3'
Arabidopsis centromere	5'-TTGAGAAGCAAGAAGAAGGT-3'
Human centromere	5'-AGAATCTGCAAGTGGATATT-3'

Video S1 Time-lapse microscopy visualizes the dynamics of the dCas9-RNA complex to label the telomeres of fixed *N. benthamiana* nuclei. During ~10 min the telomere signals (red puncta) appear and increase. In addition, a certain background labelling within the nuclei occurs (see also fig. 5).
